# Sub-carbonate of Iron in the Treatment of Uterine Hemorrhage

**Published:** 1846-03

**Authors:** S. Malherbe

**Affiliations:** Bonvillard, Switzerland


*Sub-Carbonate of Iron in the treatment of Uterine Hemorrhage.* By S. Malherbe, M. D., of Bonvillard, Switzerland. (Translated for this Journal, from the Journal des Connaiscances Medico-Chirurg., October, 1845.)—Why have authors classed the preparations of Iron amongst emmenagogues? Doubtless because they have regarded them as exerting a powerful stimulation on the uterine system, the effect of which would be to provoke menstruation. The result of observations on this point would lead us to expect from their use considerable hemorrhage. Hence, it was not until we had read the learned treatise of Therapeutics and Materia Medica by Professors Trousseau and Pidoux, (article Iron, class Tonics,) in which these authors cite the case of a chlorotic female affected with abundant uterine hemorrhage, and which was successfully treated with lozenges of ferruginons chocolate, that we ventured to use the therapeutic agent in question. We confess that we apprehended effects opposite to those we desired; but our fears were dispelled, and the result has surpassed our hopes, as may be seen by the subjoined cases.

CASE I. P——, a mechanic, consulted me in January, 1842, in relation to his wife, who was 42 years of age; strong, robust, of a rather lymphatic temperament, who had menstruated regularly and enjoyed good health, with the exception of a slight uterine hemorrhage which occurred in November, 1841. He stated to me that his wife was suffering great loss of blood and was very feeble—that the hemorrhage was preceded by pain in the lower abdomen and loins, &c. She was, however, still able to attend to her household affairs. We prescribed as follows:

℞. Pulv. sub-carbonate of Iron, 155 grs.
Pulv. cinnamon, - - - - 10 "

Mix, and divide into twenty doses—one to be taken thrice daily in a little sweetened water.

In two days the hemorrhage ceased. She was directed to take the remainder of the doses in order to ensure success, and has had no further return of the disease. Regimen was tonic and analeptic.

CASE II. B——, aged 48 years, had menstruated regularly, the mother of three children, of a good constitution, and in the enjoyment of fine health, was taken without known cause, with great uterine hemorrhage. I saw her on the 13th May, 1843, and found her in bed, pale and feeble, with considerable flow of blood. She had been suffering several days with pain in the loins and hypogastric region—was agitated, could not sleep quietly; pulse irregular and frequent; occasional chills. I ordered her:

℞. Pulv. sub-carb. Iron, 180 grs.
Pulv. cinnamon, - 7 "

Mix, and divide into fifteen doses—one to be taken thrice daily in a little tea. Perfect repose. Cooling drinks.

On the fourth day the hemorrhage had entirely ceased. The patient took the remaining doses and continued well. In July following, she consulted me for the same affection. I ordered the same prescription, and with the same success.

CASE III. On the 23d September, 1843, I was called to see Miss P——, aged 25 years, of a sanguineous temperament and habitual good health, although irregularly menstruated. On the 26th she suffered hemorrhage, and on the 28th was compelled to take her bed. I found her feeble, pale, with difficult respiration, palpitations, small and frequent pulse, thirst, and pains in the loins and abdomen. The flow was considerable, and increased by motion; no sleep; agitation; apprehension. I prescribed:

℞. Pulv. sub-carb. Iron, 210 grs.
Pulv. cinnamon, - 7 "

Mix, and divide into twenty doses—one to be taken every three hours, in sweetened water. Perfect rest. Crowfoot tea and broth.

30th Sept. Same state—treatment continued. Oct. 1st. Dry cups to the chest; flow suspended an hour after, but returned during the night. 2nd. Flow diminished—nothing but a few coagula discharged; able to sleep. 3rd. Flow arrested; general condition good; the patient can move about—same treatment continued. Convalescence was slow because the hemorrhage had been great; several weeks were required to restore her to perfect health, during which she used animal broths, vegetables, and a little red wine; she also took a tea made with *garmander* (chamedryos amarum) and orange peel. I see her frequently, and her health is perfectly good.

Case IV. On the 23d October, 1844, a female, 62 years of age, robust, who had borne several children, and ceased menstruating, consulted me for uterine hemorrhage of several weeks continuance, brought on by mental distress. She travelled a league to see me; was feeble, pale, had pains in the loins and abdomen, a small and feeble pulse, free respiration, chills, headache, &c. I recommended nourishing diet, rest, and the powders of sub-carbonate of Iron and cinnamon.

I was a long time without seeing her, but met her accidentally in the village, when she told me she had been very much relieved. As she had not taken enough of the powders, I prescribed others; for they should in such cases be continued until the disease be arrested. I have not since seen her.

Case V. B——, aged 26 years, the mother of three children, of a lymphatico-sanguine temperament, generally healthy, was delivered of her last child about a fortnight ago. I was called to see her on the 10th November, 1844, and learned that she had been taken with uterine hemorrhage three days before, without evident cause. Found her pale, with headache at night, sleepless, very feeble, suffering occasional pains in the loins and hypogastrium, thirst, anorexia, with healthy stools, cough, palpitations, small and frequent pulse, hot skin, and occasional chills—the flow was coagulated. I prescribed the same powders, rest, broth, crowfoot tea sweetened, &c. The hemorrhage was arrested on the 12th, and returned no more.

Case VI. R——, 46 years of age, unmarried; sanguineous temperament, strong, robust, was healthy until her menstruation became irregular and followed by copious hemorrhage, which left but a short interval before the return of the menses. I was first consulted by her on the 9th November, 1844: she was feeble, had good appetite, a little distress in the loins and epigastrium; pulse small and frequent, skin natural. This state had existed three weeks. I prescribed, after her menstruation, a combination of 180 grs. of sub-carb. Iron and 8 grs. of pulv. cinnamon, to be divided into sixteen doses—one of which to be taken every three hours.

Dec. 2d. General condition better—flow diminished. Prescribed 25 more doses of 15 grs. each, to be taken every two hours. 10th. Hemorrhage arrested; strength returning; powders to be continued every third hour. The cure was complete. I will add that, in this case a physician insisted that there was an organic affection of the uterus, &c.

Case VII. V——, aged 35 years, nervoso-sanguineous temperament, medium stature, had had several children, an attack of nervous fever in 1838, but generally healthy until the end of October, 1844, when she experienced pains in the abdomen and lower extremities, occasional chills, palpitations, and headaches. She was suddenly taken with severe uterine hemorrhage, which returned every two or three days. Her state on the 11th November, 1844, is as follows: Pale, restless during the night, pulse small, frequent and irregular, pains in abdomen, lungs and encephalon normal. Prescribed sub-carb. ferri and cinnamon, and quiet. A few days after, she expelled a large coagulum, containing a macerated and bloodless fœtus about three months old, which appeared to have been dead for some time. I ordered the powders to be continued in order to arrest the hemorrhage.

In this case the remedy moderated the hemorrhage, but could not arrest it so long as its cause remained. It will be remarked that the abortion was not occasioned by the Iron, for the hemorrhage existed prior to its administration.

Case VIII. On the 25th October, 1844, I was called to see Mrs. D——, aged 25 years, robust, sanguineous temperament, usually healthy, and four months pregnant. She was taken, without known cause, eight days since, with great uterine hemorrhage, preceded by the expulsion of the fœtus; when I saw her, the flow was continuing, face pale and haggard, feeble, warm skin, thirst, little appetite, palpitations, pulse small, frequent and irregular, restless sleep. I prescribed the powders. 29th. Flow diminished. 30th. Flow ceased in the morning. Powders continued every three hours to confirm the cure.

Case IX. Called on the 14th February, 1845, to see Mrs. V——, aged 27 years, lymphatic temperament, had, in 1843, ptyriasis capitis; otherwise healthy—gave birth to her first child ten days ago, and was taken, without known cause, five days afterwards with considerable uterine hemorrhage. The midwife had used some remedies without success. I found the patient feeble, pale, with lumbar pains, small and rather frequent pulse, general depression, unquiet sleep, and syncope whenever moved in bed. I ordered the powders of iron (210 grs.) and cinnnamon (8 grs.) divided into twenty doses.

The hemorrhage ceased at 11 P. M. She had commenced taking the powders at noon; one every half hour, and then every hour. Broths, herb tea, analeptics, &c., completed the cure.

Case X. C——, aged 31 years, lymphatico-sanguine temperament, good health until the end of October, 1844, but menstruated too freely. Was left quite feeble after each period. She consulted me in April, 1845. Nothwithstanding her weakness, she still had some appetite. Digestion laborious; alvine discharges natural; pulse small and frequent; sleep good; pain in loins and hypogastrium; other functions normal. I prescribed the powders in doses similar to the preceding case; one dose to be taken the two days preceding menstruation and during the three first days of its existence. The desired effect was secured; the flow was less copious, general health better, digestion improved. The remaining doses were taken before the next return of menstruation in order to regulate it. The result was satisfactory, as I had anticipated.

Case XI. On the 23d May, 1845, I was summoned to see Miss L. B.——, aged 24 years, of a sanguine temperament, robust, and of good health. She was taken in the night, without known cause, with very great uterine hemorrhage. I found her feeble, pale, agitated, rigors followed by heat, small, contracted, but resisting pulse, thirst, pain in the loins and hypogastrium, &c. The hemorrhage still continuing, I gave her the powders. On the morning of the 26th the flow was arrested, and health gradually restored.

Case XII. Mrs. M——, 27 years of age, had two children, enjoyed good health, save that the menstrual flux is excessive, and continues five days, being followed by debility and prostration. Indeed her state was rather one of hemorrhage than of menstruation. I ordered her 60 grs. carb. fer. with 8 grs. pulv. cinnamon, to be divided into four doses—one to be taken at night in a little sweetened water. The first dose diminished the flux; the second lessened it one half. My object being simply to diminish the discharge, my design was attained.

Case XIII. A female, asthmatic, was taken a fortnight after parturition, with violent uterine hemorrhage. I found it very difficult to control, by refrigerants, cold lotions to the abdomen and hands; it would always return in a few days. I was obliged to resort to the sub-carb. of iron in large doses. Since then the hemorrhage has not returned, although convalescence was very slow.

In 1839, I saw a lad 13 years of age, feeble, and who suffered with epistaxis every day for about an hour, which left him weak and dejected, and finally caused his death. Had I then known the use of the sub-carbonate, he might have been saved.

In 1844, I attended Mrs. D——, 32 years of age, of a sanguine temperament, who had been bleeding at the nose several days. A physician thought her case fatal, such was her state of anæmia. Her blood was pale, for she had lost four or five pints of it. I ordered her 180 grs. sub-car. fer. and 15 grs. pulv. cinnam., to be divided into twenty doses—one of which to be taken every two hours. The first doses arrested the epistaxis. All known remedies had been previously used in vain.

The details of my cases may appear too scanty; but I have furnished only the prominent facts, in order not to lengthen this communication. These cases demonstrate that the subcarbonate of Iron is a good hæmostatic remedy in uterine hemorrhages, even in those which proceed from the presence of the ovum or its appendages. (See case VII.) In all the other cases, the flow ceased in two or three days, and in one of them in ten hours.

The 10th and 12th cases will show that the remedy may be useful in excessive menstruation; that it may moderate and even regulate it. They all teach us that it can be used without hazard. I know that we should not be too exclusive; but, for my part, I prefer this preparation because it is not disagreeable when taken with a little cinnamon. It is necessary to have a good article. M. Bertholet, apothecary at Grandson, has prepared it for me very skilfully, and it always acts satisfactorily. It will be remarked, that the doses do not vary much, and that they were given at greater or less intervals, according to effect. We need no longer be afraid to give it in acute uterine hemorrhage, since it is not an emmenagogue as stated in works on Materia Medica. Nothing but observation could have convinced me of its opposite effects. I am therefore authorized to state that the sub-carbonate of Iron is not an emmenagogue, but, on the contrary, arrests and moderates the menstrual flux—will arrest internal hemorrhages, and, in short, is a powerful hæmostatic agent. Let this active agent be no longer dreaded. I furnish these cases in the hope that they may prove useful to that extensive class of practitioners whose first object is the relief of their patients. —*Southern Med. and Surg. Jour.*

